# Evaluation of Residual Compressive Strength and Behavior of Corrosion-Damaged Carbon Steel Tubular Members

**DOI:** 10.3390/ma11071254

**Published:** 2018-07-20

**Authors:** Jin-Hee Ahn, Seok-Hyeon Jeon, Young-Soo Jeong, Kwang-Il Cho, Jungwon Huh

**Affiliations:** 1 Department of Civil Engineering, Gyeongnam National University of Science and Technology, Jinju, Gyeongnam 52725, Korea; jhahn@gntech.ac.kr (J.-H.A.); jshyeon1950@gmail.com (S.-H.J.); 2 Seismic Simulation Test Center, Pusan National University, Yangsan, Gyeongnam 50612, Korea; ysjung@pusan.ac.kr; 3 R&D Team, Tekhan Inc., 601, 324, Gangnam-daero, Gangnam-gu, Seoul 06252, Korea; kwangcho222@gmail.com; 4 Department of Civil & Environmental Engineering, Chonnam National University, Yeosu, Jeonnam 59626, Korea

**Keywords:** residual compressive strength, tubular member, cross-sectional damage, corrosion, compressive loading test, FEA

## Abstract

Local corrosion damage of steel structures can occur due to damage to the paint-coated surface of structures. Such damage can affect the structural behavior and performance of steel structures. Compressive loading tests were, thus, carried out in this study to examine the effect of local corrosion damage on the structural behavior and strength of tubular members. Artificial cross-sectional damage on the surface of the tubular members was introduced to reflect the actual corroded damage under exposure to a corrosion environment. The compressive failure modes and compressive strengths of the tubular members were compared according to the localized cross-sectional damage. The compressive loading test results showed that the compressive strengths were affected by the damaged width within a certain range. In addition, finite element analysis (FEA) was conducted with various parameters to determine the effects of the damage on the failure mode and compressive strength of the stub column. From the FEA results, the compressive strength was decreased proportionally with the equivalent cross-sectional area ratio and damaged volume ratio.

## 1. Introduction

Circular steel members have been used as major structural components in various structural systems. In energy power facilities, circular steel members have been used as pipeline or structural members of main platforms or substructures [1,2,3,4]. In offshore and maritime structures, they have been used as the main structural components for structural platforms on oilrigs, observation facilities, and wind and power plants. In addition, for infrastructures, circular steel members have been used in bridges, buildings, and plant systems, etc. Generally, paint coating has been applied to these circular members for corrosion protection, aesthetics, and sustainable maintenance. However, due to deterioration or localized damage of the paint-coated surface of circular members, localized corrosion commonly occurs on paint-coated surfaces exposed to atmospheric corrosion environments [5,6,7]. This localized corrosion damage to the surface of circular steel members can affect their structural safety, performance, and durability, because corrosion damage is related to the cross-sectional damage on the surface of the member.

The corrosion pit under a pipeline was investigated by electrochemical impedance techniques (EIS) and the corrosion behavior of iron and steel was evaluated depending on carbonate solution, chloride, and carbon dioxide [8,9,10]. Experimental and numerical studies have been conducted to examine the variation of structural performance of structural members according to cross-sectional damage induced by corrosion [11,12,13,14,15,16]. Additionally, the reinforcement or rehabilitation method has been used to examine cross-sectional damaged members [17,18]. Some studies have considered the ideal corrosion model for examining even cross-sectional damage on structural member surfaces. To consider the irregular cross-sectional damaged surfaces due to corrosion, compressive loading tests have been conducted on inclined and vertical circular steel members with both uneven and even cross-sectional damage. In these tests, damage was artificially induced by a mechanical process and hand drills to compare the differences between the compressive resistances and cross-sectional damage condition caused by corrosion [19].

In these previous studies, half- and fully-damaged cases of cross-sectional corrosion of the outer circumference of circular steel members [19] were considered. Based on the decreased compressive strength ratios from these compressive loading test results, an equation of residual compressive factors was suggested, depending on the cross-sectional damage ratio [20]. However, it was difficult to determine the effects of local cross-sectional damage caused by corrosion on the compressive behaviors of the members, since relatively widely distributed cross-sectional damage and full damage of the circumference occurred on the surface of the tubular section, as discussed in the previous study [19,20]. The major variables affecting the compressive strength and behavior of the structural members were not able to be determined.

In this study, artificial cross-sectional damages with various damaged widths and heights were induced on the surface of tubular specimens to examine the major variables that affect the compressive behaviors of circular tubular members. After compressive loading tests of the circular tubular specimens, their compressive failure modes and compressive resistant capacities were evaluated and compared. To more clearly evaluate their compressive resistant capacities, FEA was additionally conducted to determine the parameters of various cross-sectional damages. Using a range of FEA models, the major variables that affect the compressive strength of the specimens were found and it was, therefore, not necessary to use a large number of specimens for the experiments. A total of 120 FEA models were constructed and used for analysis in this study. After performing the analysis, the failure modes were checked to determine the major factors and the compressive strength values of the members were evaluated to determine the variables affected by the damages. Finally, the effect of the localized cross-sectional damage condition on the compressive resistance of the circular tubular members was quantitatively evaluated.

## 2. Compressive Test Conditions

### 2.1. Localized Cross–Sectional Damaged Tubular Specimens

**Figure 1:**
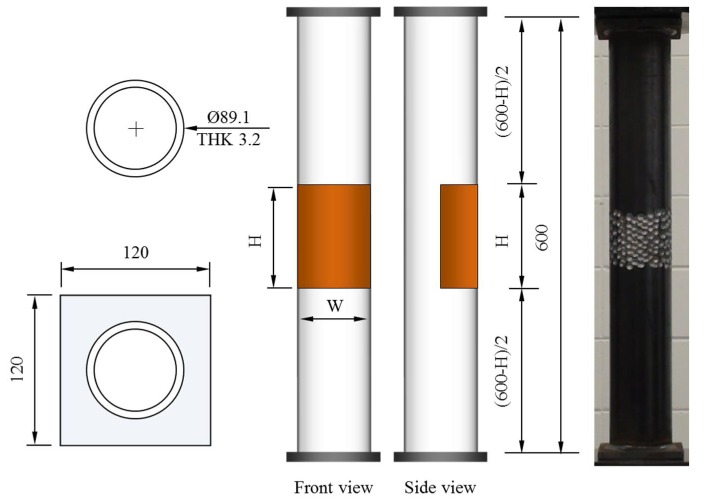
Dimensions of the compressive specimens (unit: mm).

To consider the various cross-sectional damaged conditions on the surface of the circular tubular members compared to those on the specimens used in the previous study [19,20], a small circular tubular pipe 89.1 mm diameter (thickness: 3.2 mm) was selected for the compressive loading test specimens. Since the dimensions of the tubular pipe can differ from the official dimensions from the fabrication process, the tubular pipe dimension was measured and its actual diameter was 90 mm. Circular tubular specimens were cut to a 600 mm height and steel plates of 120 mm width and 10 mm thickness were attached to each end of the tubular specimen as shown in Figure 1. These circular tubular specimens were designed as stub columns with a slenderness ratio (λ) of 9.87. The steel grade of the tubular specimens was STK 400, having a yield stress of over 216 MPa and a tensile strength of over 400 MPa.

In this study, the yield stress was chosen to be 310 MPa in accordance with the material test. To replicate the corrosion damage occurring in certain areas of the section in the tubular member, artificial damages with continuous pitting shapes were induced on the surface of the tubular specimens using a hand drill with a 12 mm diameter drill bit. Artificial cross-sectional damage condition was determined considering relative ratio to the diameter for circular specimen. Thus, a total of 18 specimens were fabricated according to the abovementioned artificial cross-sectional damage conditions.

To investigate the major variables for the compressive behaviors of the specimens, the width of the artificial cross-sectional damage of specimen was changed from D/3 (about 30 mm) to 1.6D (about 150 mm) and the height was also changed from D/3 (about 30 mm) to 1.0D (about 90 mm), as shown in Table 1. Table 1 summarizes the compressive loading test specimens. In the fabrication process, the weight of each specimen was also measured to check the cross-sectional damage before and after inducing artificial damage to the tubular specimens. In particular, in order to examine the uncertainty of the artificially damaged section, CD-H7.5W series specimens having an artificial damage with a height of 75 mm were fabricated. The artificial cross-sectional damage was not calculated at the design level of the specimen, and the weight loss was only measured after the contrived cross-sectional damage. Thus, the cross-sectional losses of the CD-H7.5W series specimens were not quantitatively dependent on the cross-sectional damaged condition, unlike those of the other specimens. From their measured weight loss, the equivalent cross-sectional areas of the specimens were also calculated for the artificial damaged region of the specimens.

From the weight loss after the artificial cross-sectional damage, the equivalent damaged thickness of the tubular member was also calculated along the damaged width of the tubular specimen. For the names of the specimens in Table 1, “C” refers to the compressive test, “R” is the reference, “D” indicates a damaged specimen, “W” indicates the width of the damage, and “H” indicates the height of the damage. The number following “W” and “H” represents the damaged ratio determined from the diameter of the circular specimen. Thus, specimen CD-H3W3 is the compressive test specimen with an artificial damage of 30 mm height and 30 mm width.

### 2.2. Loading Test Conditions

**Figure 2:**
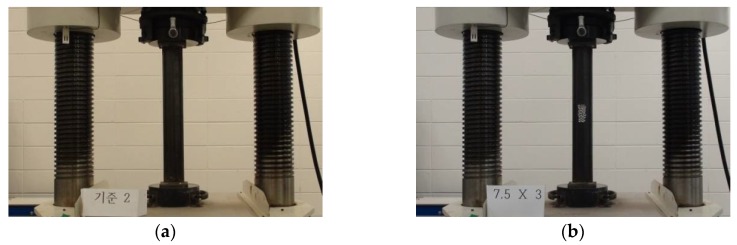
Experimental setup of compressive loading test specimen: (**a**) CR-H0W0-2 specimen; and (**b**) CR-H7.5W3 specimen.

To examine the compressive resistant behaviors of the tubular specimens with localized cross-sectional damage, an electric hydraulic servo system (Shimadzu, Kyoto, Japan) with a 5000 kN capacity universal testing machine (UTM) was used to apply compressive loading to the specimens. To control the compressive load, displacement load control was used at a rate of 1.5 mm/min. Compressive loading was applied to the test specimens until the occurrence of compressive failure, such as a local buckling failure. During compressive loading to the tubular specimens, their compressive behaviors were recorded using a video camera to observe the failure behaviors depending on the applied load level. Figure 2 shows the test setup of the compressive loading test for the CR-H0W0-2 and CR-H7.5W3 specimens.

## 3. Test Result Evaluation

### 3.1. Failure Modes

**Figure 3:**
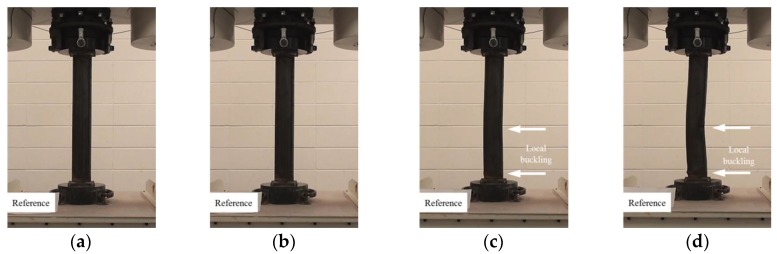
Compressive failure process of specimen CR-H0W0-1: (**a**) before load; (**b**) at yield load; (**c**) at compressive strength; and (**d**) at final failure.

In this study, the localized artificial cross-sectional damage determined from the relative ratio of the diameter of the tubular specimen was considered to investigate the major variables affecting compressive behaviors related to failure modes and the variation of compressive strengths. It was found that the failure modes could be affected by major variables, such as height, width, and damaged depth related to their cross-sectional damaged condition. Thus, the damaged width and height were changed from D/3 (about 30 mm) to 1.6D (about 150 mm for width) and 1.0D (about 90 mm for height). After compressive loading tests, the failure modes among the CR-H0W0 series specimens were compared to determine the specified failure modes. Figure 3 shows the compressive behaviors of the representative tubular specimens depending on the applied load level (at yield load, at compressive loading, and at final failure).

**Figure 4:**
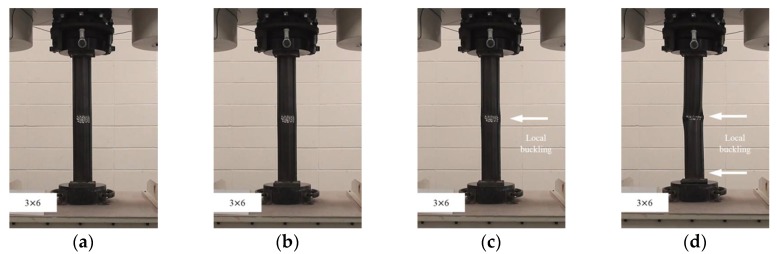
Compressive failure process of specimen CR-H3W6: (**a**) before load; (**b**) at yield load; (**c**) at compressive strength; and (**d**) at final failure.

**Figure 5:**
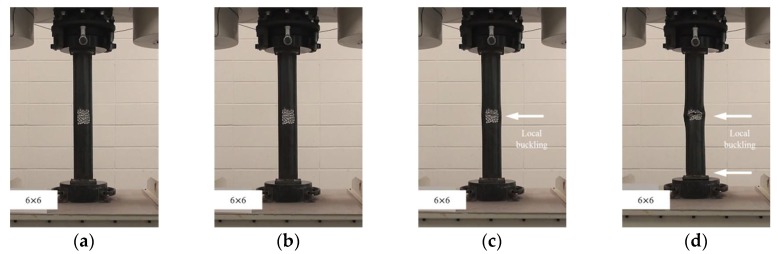
Compressive failure process of specimen CR-H6W6: (**a**) before load; (**b**) at yield load; (**c**) at compressive strength; and (**d**) at final failure.

**Figure 6:**
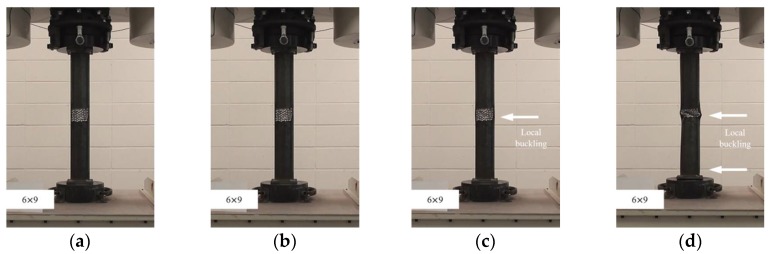
Compressive failure process of specimen CR-H6W9: (**a**) before load; (**b**) at yield load; (**c**) at compressive strength; and (**d**) at final failure.

**Figure 7:**
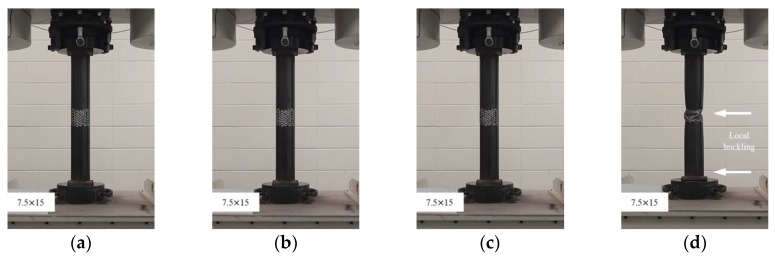
Compressive failure process of specimen CR-H7.5W15: (**a**) before load; (**b**) at yield load; (**c**) at compressive strength; and (**d**) at final failure.

**Figure 8:**
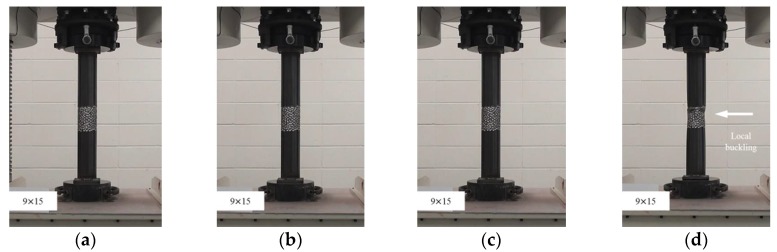
Compressive failure process of specimen CR-H9W15: (**a**) before load; (**b**) at yield load; (**c**) at compressive strength; and (**d**) at final failure.

The failures of the tubular specimens without artificial cross-sectional damage were initiated by local buckling near the loading plate, which was accompanied by lateral buckling of the tubular specimen after the initial local buckling, as shown in Figure 3a–d. On the other hand, the tubular specimens with artificial cross-sectional damage showed various final local buckling failure modes that were squashed or folded in the artificial damaged section depending on their cross-sectional damaged condition as shown in Figure 4, Figure 5, Figure 6, Figure 7 and Figure 8. In the case of the CR-H3W6 specimen, with a relatively lower cross-sectional damage height, as shown in Figure 4, a local buckling failure mode was found with the fold in the artificial cross-sectional damage region. In the case of specimens with a certain cross-sectional damage height of 60 mm and width of 60 mm, a local buckling failure mode with unusual deformation (such as shear failure mode of thin plate) was observed due to the cross-sectional damaged condition, as shown in Figure 5 and Figure 6. For the specimen with the wider cross-sectional damage of 1.6 D (about 150 mm for width), the buckling failure mode showed irregularly crushed failure in the artificially damaged section as shown in Figure 7 and Figure 8.

These differences between failure modes could be due to the cross-sectional resistance and stress distribution on the surface of the damaged section caused by localized corrosion damage. From these failure modes, it can be assumed that the cross-sectional damaged shape and condition can be influenced by various forms of compressive failure modes that are affected by localized corrosion damage.

### 3.2. Compressive Loading Results

#### 3.2.1. Summary of Compressive Loading Tests

**Figure 9:**
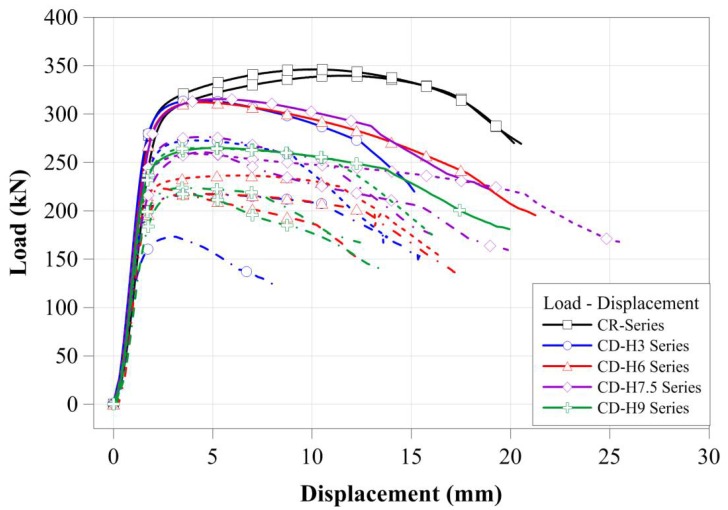
Compressive load-displacement relationships of all specimens.

In order to quantitatively compare the change in the compressive strengths of circular tubular member specimens according to the localized cross-sectional damage condition, the compressive load-displacement relationships of the specimens are presented in Figure 9. All compressive loading test specimens showed typical compressive loading resistance behaviors, as shown in Figure 9. The CR-H0W0 series specimens showed a relatively larger ductile behavior after yield loading than that of the tubular member specimen with artificial cross-sectional damage. For the tubular member specimen with artificial cross-sectional damage, compressive displacements relatively decreased from yield load to failure; this tendency was greatly affected by the specimens with the higher cross-sectional damage as shown in Figure 9. The compressive loads of the tubular member specimens with artificial damage to the sections affected the cross-sectional resistance condition, as determined by the cross-sectional damaged level. Thus, it is inferred that the compressive loads decreased according to the cross-sectional resistance condition determined from the cross-sectional damaged level. Table 2 summarizes the maximum compressive load of each test specimen to quantitatively show the compressive load and displacement relationships.

**Figure 10:**
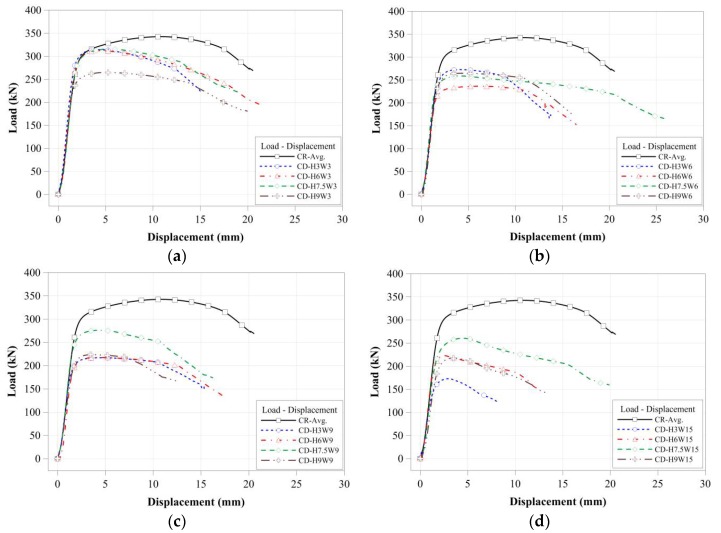
Compressive load-displacement relationships depending on cross-sectional damaged width: (**a**) 30 mm damaged width; (**b**) 60 mm damaged width; (**c**) 90 mm damaged width; and (**d**) 150 mm damaged width.

**Figure 11:**
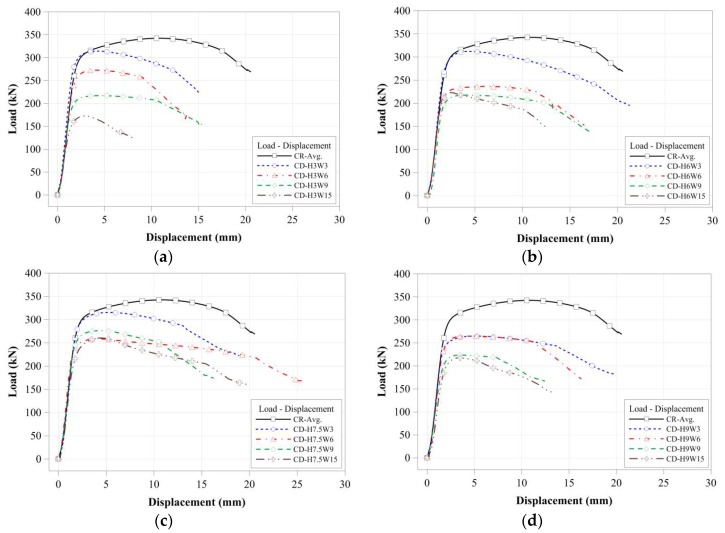
Compressive load-displacement relationships depending on cross-sectional damaged height: (**a**) 30 mm damaged height; (**b**) 60 mm damaged height; (**c**) 75 mm damaged height; and (**d**) 90 mm damaged height.

As shown in Figure 9, the compressive strengths of the tubular members with artificial cross-sectional damage were affected by the cross-sectional damaged conditions. To compare the effect of the cross-sectional damaged width of the tubular members on their compressive behavior, the compressive load-displacement relationships of the tubular members were compared separately in accordance with the cross-sectional damaged condition, as derived from Figure 10a–d and Figure 11a–d.

#### 3.2.2. Effect of the Cross-Sectional Damaged Condition on Compressive Strength

**Figure 12:**
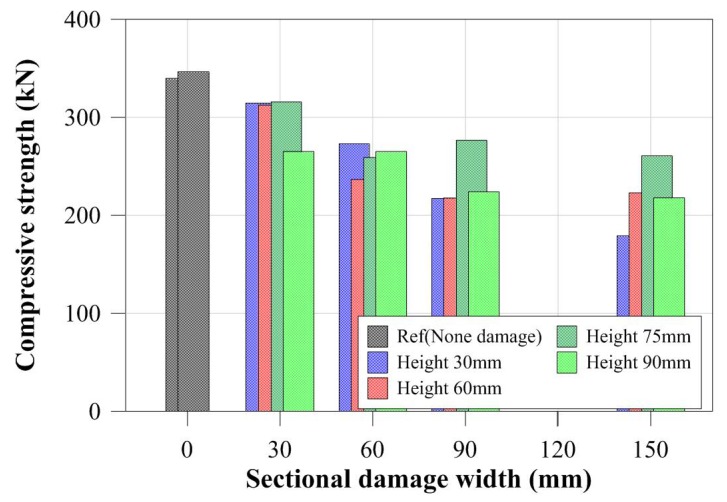
Compressive strength depending on sectional damaged width.

For specimens with the same cross-sectional damaged width, the compressive strengths were determined from the certain cross-sectional damaged width within a certain range as shown in Figure 10. For the specimens with a greater cross-sectional damaged width, as shown in Figure 10d, the compressive strengths were more strongly affected by the cross-sectional damaged height than by the cross-sectional damaged width. Their compressive behaviors were also shown to concur with the change in their compressive strength. Thus, for specimens with different cross-sectional damaged widths ranging from 30 mm to 90 mm, their compressive behaviors from yield load to failure were relatively similar to those of specimens with the same cross-sectional damaged widths. Conversely, for the specimen with a 150 mm damaged width, the compressive behaviors changed depending on their cross-sectional damaged height. Figure 12 summarizes the compressive strength of each tubular specimen depending on the artificial cross-sectional damaged width. The compressive strength of the tubular specimen series with the same artificial cross-sectional damaged width was slightly decreased compared to that of the specimen series with no cross-sectional damage, except for the CD-W15 series, since their compressive strengths were determined from their cross-sectional damaged width within a specific range. However, their critical value was not clear.

**Figure 13:**
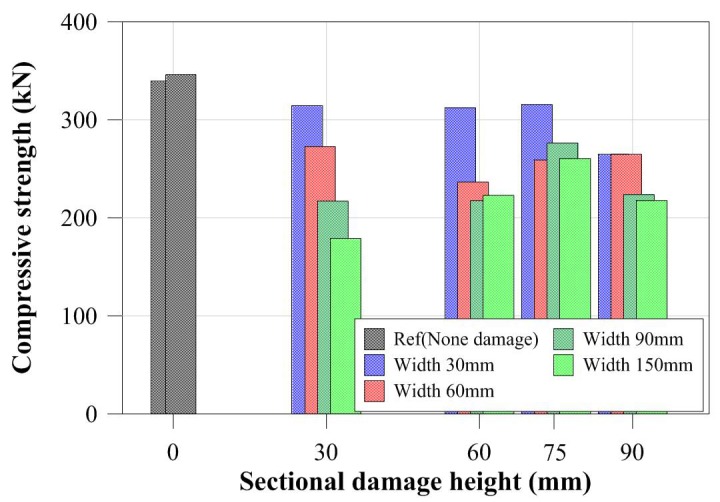
Compressive strength depending on cross-sectional damaged height.

Figure 11 shows that the compressive strengths and behaviors of specimens were compared to those of specimens with the same cross-sectional damaged. The compressive strength of CD-H3W15 specimen was decreased by compared to CD-H3W3 which is affected by the cross-sectional damaged width for the same damaged height condition as shown in Figure 12. Figure 13 summarizes the compressive strength of each tubular specimen depending on the artificial cross-sectional damaged height. In the compressive strength of the tubular specimen series with the same artificial cross-sectional damaged height, the compressive strengths decreased depending on each cross-sectional damaged condition. For specimens with relatively lower cross-sectional damaged height, the difference between the compressive strength of each specimen decreased, as shown in Figure 13. In the case of the specimens with lower cross-sectional damaged height (less than 60 mm in damaged height), the compressive strength decreased, which affected the cross-sectional damaged width.

### 3.3. FEA of Compressive Loading Cases

#### 3.3.1. FEA Model and Parameters

**Figure 14:**
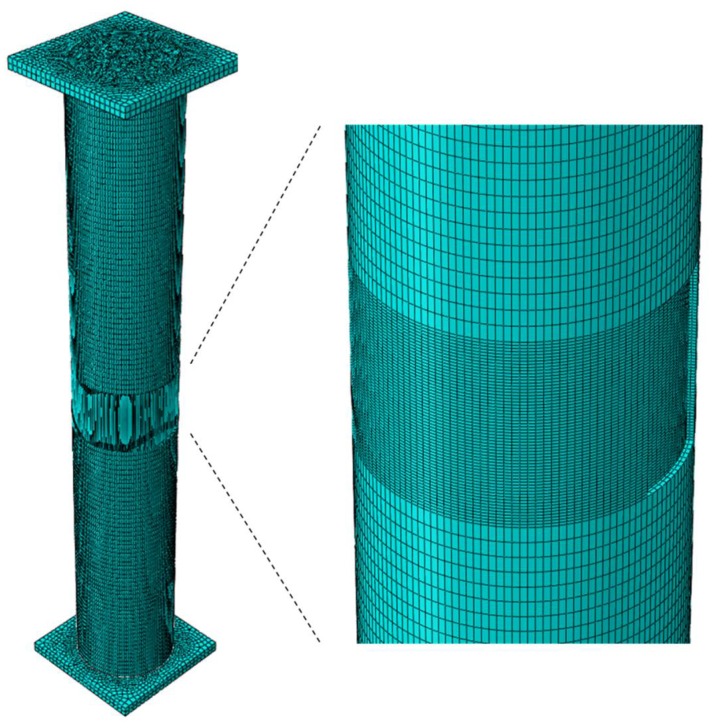
FEA model.

In order to compare the compressive strength of the stub steel tubular member in the test result and determine the factors that affect the compressive strength of stub tubular members with cross-sectional damage, their compressive strengths were examined using the FEA program Abaqus 6.14. The FEA model was made with the same dimensions as the model for the loading tests; thus, the model has three components: a pipe of 600 mm length with a diameter of 89.1 mm and a thickness of 3.2 mm, and two 120 mm rectangular plates with a thickness of 10 mm. The FEA model simulated in this research was fabricated of steel with a modulus of elasticity of 205 GPa and Poisson’s ratio of 0.3. The yield stress value was selected to be 310 MPa, based on the material tests. The bilinear isotropic hardening behavior of this model was made to simulate the plastic behavior of the material tested. In addition, the plates were rigid plates to avoid deformation. In this model, displacement was applied at the center of the top plate to simulate the real loading test. In addition, the boundary condition was considered to ensure the simulation was as close to the experiment as possible. The bottom plate was defined as the fixed boundary condition and the lateral displacement of the top plate was restrained. Figure 14 shows the FEA model for this study.

To simulate and identify the compressive strength values and behaviors of the specimens, their cross-sectional damage parameters were selected. A range of models were developed with different corroded depths, heights, and widths: the corroded depth varied from 0.5 mm to 2 mm with a 0.5 mm increment, the corroded height varied from 60 mm to 300 mm with a 60 mm increment, and the corroded width varied from 28 mm to 280 mm with a 28 mm increment. Similar to the compressive loading test specimens, the name of the FEA model was determined such that “C” refers to the compressive test, “D” refers to a damaged specimen, “W” indicates the damaged width, and “H” indicates the damaged height. The value after “CD” refers to the damaged thickness of the FEA model. Thus, the CD0.5-H60W28 model refers to a compressive analysis model with an artificial damage of 0.5 mm thickness, a 60 mm height, and a 28 mm width. Table 3 summarizes the properties of the FEA models.

#### 3.3.2. FEA Model and Parameters

**Figure 15:**
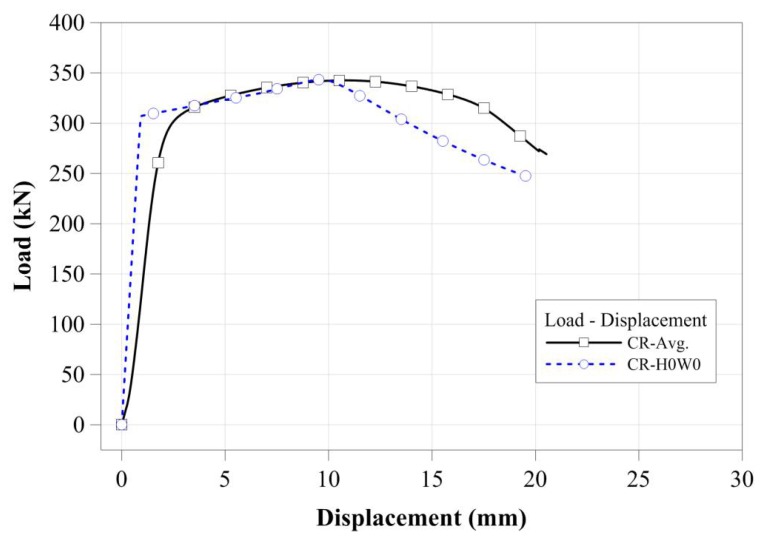
Comparison of the FEA result and the test result.

To verify the FEA model, the compressive loading test result was compared to the result of the FEA model. It is difficult to develop an FEA model for a compressive loading test specimen with irregular cross-sectional damage. The CR-H0W0 specimen without cross-sectional damage was modeled and its load-displacement curves were compared. For this comparison, the measured dimensions were applied to the FEA model. Figure 15 shows the load-displacement comparison for the CR-H0W0 specimen. As shown in Figure 15, its ultimate strength and yield load were similar to those of the test results and the tendency of the load-displacement curve also agreed with that of the test results. Thus, the compressive strength and behavior of the stub compressive tubular members with local cross-sectional damage by corrosion can be examined using this model.

**Figure 16:**
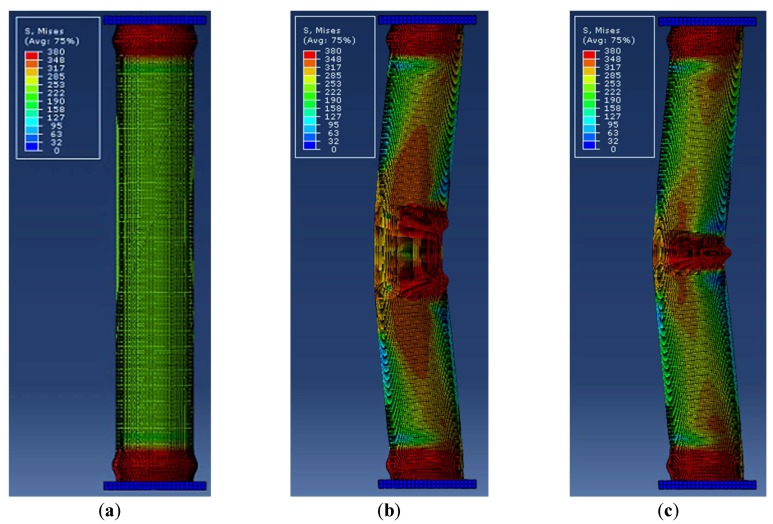

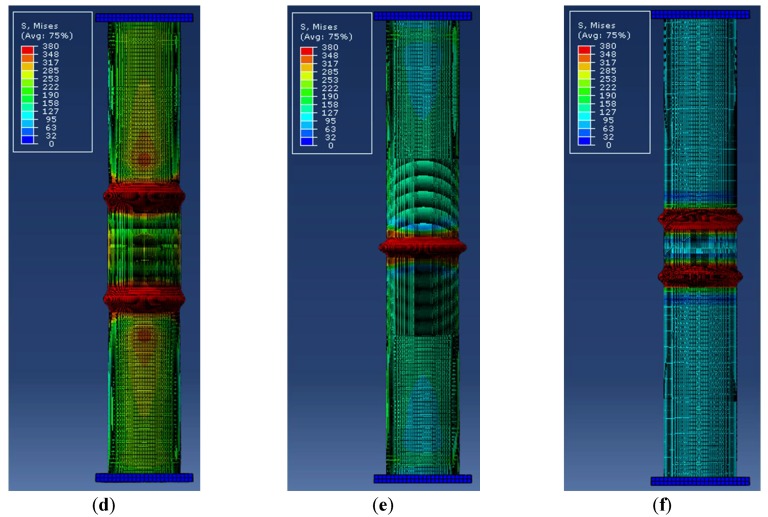
Representative failure modes from FEA: (**a**) failure mode 1; (**b**) failure mode 2; (**c**) failure mode 3; (**d**) failure mode 4 (**e**); failure mode 5; and (**f**) failure mode 6.

In this study, tubular specimens with localized artificial cross-sectional damage were tested for their cross-sectional damage ratio for their diameter. The loading test results showed that squashed or folded failure modes occurred in the artificially-damaged section. The FEA results showed that the compressive failure modes were similar to the six failure cases shown in Figure 16. To determine the failure mode, local buckling failure and lateral buckling failure were considered from each FEA model result. In failure mode 2, lateral buckling failure occurred and two local buckling failures were observed at the cross-sectional damage boundary. In failure mode 3, lateral buckling failure occurred and one local buckling failure was observed at the cross-sectional damage section. In failure mode 4, two local buckling failures were observed at the damaged boundary, without lateral buckling. In failure mode 5, only one local buckling failure was observed at the cross-sectional damage section. Failure mode 6 as similar to that of failure mode 4, but was determined by other factors (cross-sectional damage condition) than those for failure mode 3. The failure modes of the specimen were affected by their damaged condition, such as the damaged height and damaged width. Their damaged depth was also affected by their failure mode.

In the case of a relatively higher cross-sectional damaged model with lower damaged depth, their failure modes showed that it might be due to Failures 2 or 3. For higher damaged depth modes, the failure modes could be due to Failures 4 and 5. In particular, Failure 6 occurred in the fully-damaged model. However, because it was difficult to select the critical damage case to determine the failure mode of the specimen, the critical damage case was determined by the damaged condition and boundary condition from the compressive loading test results. However, it can be concluded that local buckling failure accompanied by lateral buckling failure, as well as local buckling failure occurred in the damaged region.

**Figure 17:**
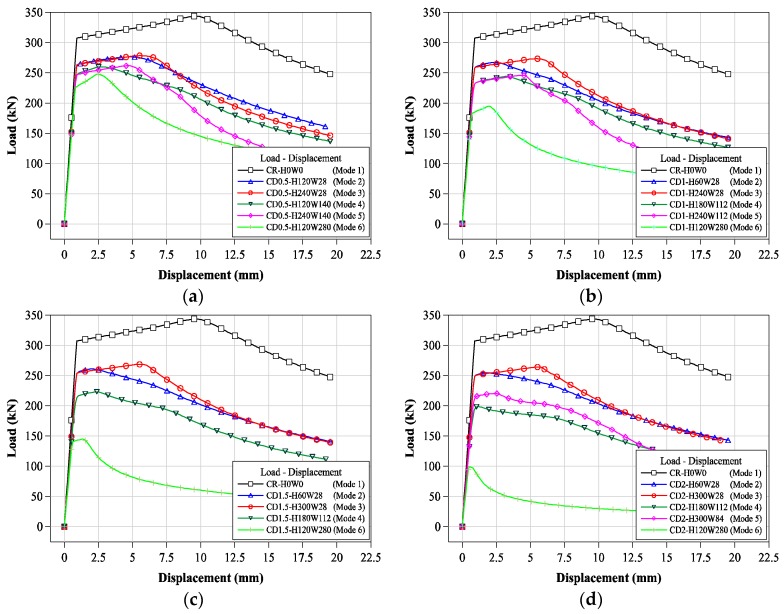
Load-displacement relationship of each corroded depth specimen: (**a**) 0.5 mm corroded depth; (**b**) 1.0 mm corroded depth; (**c**) 1.5 mm corroded depth; and (**d**) 2.0 mm corroded depth.

To examine the compressive behaviors, the load-displacement relationships were compared depending on their damaged depth, as shown in Figure 17. These load-displacement relationships were selected from the compressive failure modes. As shown in Figure 17, the compressive behaviors and strengths (yield loads) were affected by their damaged depth and condition. Their compressive yield load and strengths decreased with the damaged depth. For the same damaged depth, the compressive yield load and strengths distinctly changed based on their failure modes and compressive behaviors. For a greater damaged depth, their displacement behaviors showed that it was not clear and was shorter, without ductile behaviors. From these load-displacement relationships, the FEA results of the tubular members with localized cross-sectional damage are summarized in Table 4 as yield loads, compressive strengths, and failure modes.

**Figure 18:**
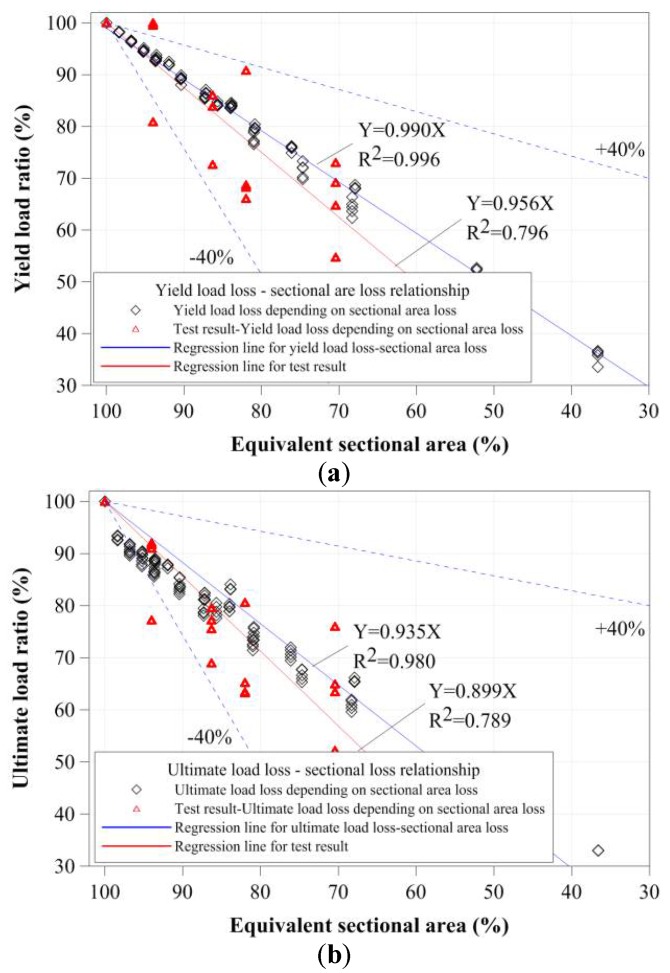
Yield strength and compressive strength depending on damaged area: (**a**) yield load ratio-equivalent damaged area; and (**b**) the ultimate load ratio-equivalent damaged area.

**Figure 19:**
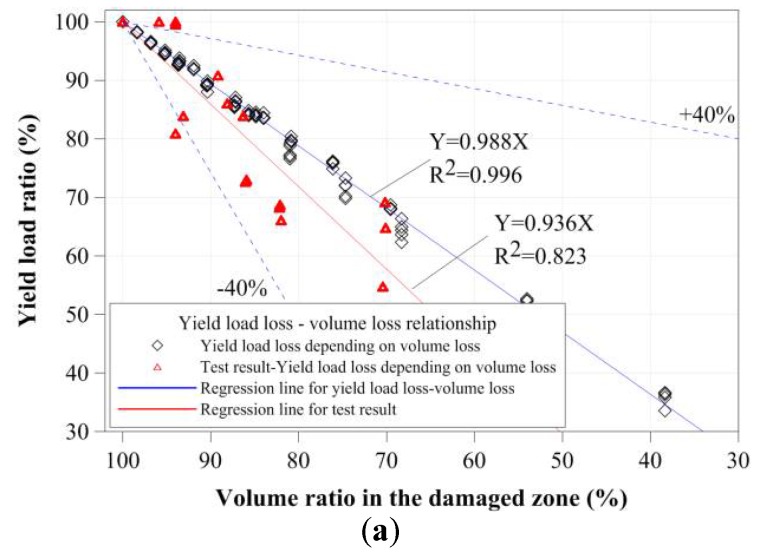

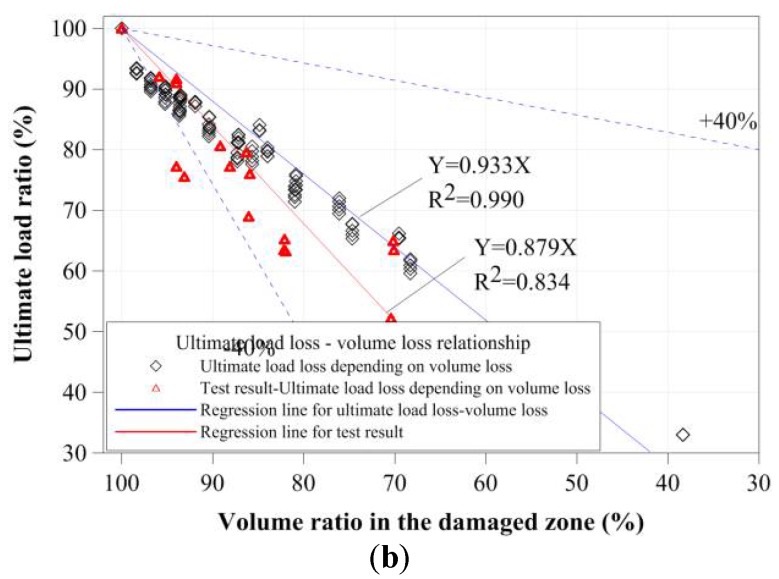
Yield strength and compressive strength depending on the volume of the damaged zone: (**a**) yield load ratio-volume ratio in the damaged zone; and (**b**) the ultimate load ratio-volume ratio in the damaged zone.

To evaluate the relationships between the cross-sectional damage and compressive strength of small tubular members, their yield loads and compressive strengths were evaluated based on the equivalent cross-sectional area ratio and damaged volume ratio from the test results, as shown in Figure 18 and Figure 19. To determine the main factor affecting compressive strength, other cross-sectional properties of the tubular member were also considered. However, the two values were similar to the decrease in the yield loads and compressive strengths of the stub compressive tubular members with local cross-sectional damage by corrosion. As shown in Figure 18 and Figure 19, the yield loads and compressive strengths were proportionally decreased depending on the equivalent cross-sectional area ratio and damaged volume ratio. However, the test results were distributed to decrease about 40% of the regression line for the FEA results, and it was shown to be effective by the minimum residual thickness and corroded shape. The yield loads decreased according to the equivalent cross-sectional area ratio and volume ratio. The compressive strengths also constantly decreased from a compressive strength of 95%, depending on their cross-sectional area ratios and volume ratios.

## 4. Conclusions

This study examined the structural behavior and strength of stub tubular steel members with localized cross-sectional damage. To consider the localized cross-sectional damage caused by corrosion, compressive loading tests and numerical analyses of stub circular members were conducted. For the compressive loading test, artificial cross-sectional damages, with various damaged widths and heights, were induced on the surface of the tubular specimens to examine the major variables that affect the compressive behaviors of circular tubular members. For numerical analyses, FEA was conducted to clarify the compressive resistant capacities of the circular tubular specimens for various damaged parameters, such as corroded depth, damaged width, and damaged height. A total of 120 FEA models were analyzed to investigate the major variables of compressive strength with cross-sectional damage by corrosion.

The compressive loading test results showed that the compressive strengths were affected by the damaged width within a certain range. The effect of compressive strength according to the damaged height was greater than that according to the damaged widths. Thus, the tendencies of yield load and failure on the same damaged width under lower damaged width were similar; however, those of the greater damaged width changed depending on the damaged height. To verify the FEA, the compressive strengths (yield loads) affected by the damaged depth and condition were determined. The compressive yield load was decreased to change the failure modes depending on the damaged depth. To clarity the compressive strength of the circular tubular member according to cross-sectional damage, the equivalent cross-sectional area ratio and damaged volume ratio were calculated. The compressive strength was decreased proportionally with the equivalent cross-sectional area ratio and damaged volume ratio.

## Figures and Tables

**Table 1:** Summary of the tubular specimen with localized cross-sectional damage.

Specimens	Damaged Height (H)/Width (W) (mm)	Weight Loss after Artificial Cross-Sectional Damage (g)	Equivalent Cross-Sectional Area (mm^2^)/(%)	Artificial Cross-Sectional Damaged Volume (mm^3^)
CR-H0W0-1	-	0	990.2/100	0
CR-H0W0-2	-	0	990.2/100	0
CR-H0W0-3	-	0	990.2/100	0
CR-H0W0_mean	-	0	990.2/100	0
CD-H3W3	30/30	14	930.7/93.99	1783.4
CD-H3W6	30/60	32	854.3/86.28	4076.4
CD-H3W9	30/90	42	811.8/81.99	5350.3
CD-H3W15	30/150	69	697.2/70.41	8789.8
CD-H6W3	60/30	28	930.7/93.99	3566.9
CD-H6W6	60/60	65	852.2/86.07	8280.3
CD-H6W9	60/90	83	813.9/82.2	10,573.2
CD-H6W15	60/150	139	695/70.19	17,707.0
CD-H7.5W3	75/30	24	949.4/95.88	3057.3
CD-H7.5W6	75/60	40	922.2/93.14	5095.5
CD-H7.5W9	75/90	63	883.2/89.2	8025.5
CD-H7.5W15	75/150	82	850.9/85.94	10,445.9
CD-H9W3	90/30	42	930.7/93.99	5350.3
CD-H9W6	90/60	83	872.7/88.14	10,573.2
CD-H9W9	90/90	125	813.2/82.13	15,923.6
CD-H9W15	90/150	209	694.3/70.12	26,624.2

**Table 2:** Summary of test results of the tubular specimen with localized cross-sectional damage.

Specimens	Damaged Height (H)/Width (W) (mm)	Damaged Volume (mm^3^)	Converted Cross-Sectional Area (mm^2^)	Compressive Strength (kN)	Displacement (mm)
CR-H0W0-1	-	0	990.2/100	0	12.0
CR-H0W0-2	-	0	990.2/100	0	10.0
CR-H0W0-3	-	0	990.2/100	0	10.9
CR-H0W0_mean		0	990.2/100	0	4.2
CD-H3W3	30/30	1783.4	930.7/93.99	1783.4	4.0
CD-H3W6	30/60	4076.4	854.3/86.28	4076.4	4.7
CD-H3W9	30/90	5350.3	811.8/81.99	5350.3	3.4
CD-H3W15	30/150	8789.8	697.2/70.41	8789.8	4.5
CD-H6W3	60/30	3566.9	930.7/93.99	3566.9	6.3
CD-H6W6	60/60	8280.3	852.2/86.07	8280.3	4.3
CD-H6W9	60/90	10,573.2	813.9/82.2	10,573.2	2.5
CD-H6W15	60/150	17,707.0	695/70.19	17,707.0	5.3
CD-H7.5W3	75/30	3057.3	949.4/95.88	3057.3	3.9
CD-H7.5W6	75/60	5095.5	922.2/93.14	5095.5	4.3
CD-H7.5W9	75/90	8025.5	883.2/89.2	8025.5	4.5
CD-H7.5W15	75/150	10,445.9	850.9/85.94	10,445.9	5.4
CD-H9W3	90/30	5350.3	930.7/93.99	5350.3	4.3
CD-H9W6	90/60	10,573.2	872.7/88.14	10,573.2	3.6
CD-H9W9	90/90	15,923.6	813.2/82.13	15,923.6	3.6
CD-H9W15	90/150	26,624.2	694.3/70.12	26,624.2	12.0

**Table 3:** Analysis parameters of the tubular members with localized cross-sectional damage.

Parameter of Analysis Model	
Corroded depth	0.5, 1, 1.5, 2 mm
Corroded height	60, 120, 180, 240, 300 mm
Corroded width	28, 56, 84, 112, 140, 280 mm

**Table 4:** Summary of FEA results of the tubular members with localized cross-sectional damage.

Specimens	Equivalent Cross-Sectional Area (mm^2^)/(%)	Volume Ratio in the Damaged Zone (%)	Yield Load (kN)/(%)	Ultimate Load (kN)/(%)	Failure Mode
CR-H0W0	863.6/100	100	267.7/100	297.7/100	1
CD0.5-H60W28	849.6/98.39	98.39	263.1/95.01	275.4/92.51	3
CD0.5-H120W28	849.6/98.39	98.39	263.3/95.01	275.9/92.68	2
CD0.5-H180W28	849.6/98.39	98.39	262.8/95.01	277.5/93.23	2
CD0.5-H240W28	849.6/98.39	98.39	262.8/95.01	278.1/93.4	3
CD0.5-H300W28	849.6/98.39	98.39	262.8/95.01	278.1/93.43	3
CD0.5-H60W56	835.7/96.78	96.78	258.7/90.39	268.7/90.27	2
CD0.5-H120W56	835.7/96.78	96.78	258.1/90.39	269.1/90.39	2
CD0.5-H180W56	835.7/96.78	96.78	258/90.39	271.7/91.26	2
CD0.5-H240W56	835.7/96.78	96.78	258/90.39	273.3/91.8	3
CD0.5-H300W56	835.7/96.78	96.78	258.1/90.39	273.5/91.87	3
CD0.5-H60W84	821.8/95.17	95.17	254.7/86.49	265.4/89.16	2
CD0.5-H120W84	821.8/95.17	95.17	253.8/86.49	265/89.03	2
CD0.5-H180W84	821.8/95.17	95.17	253.5/86.49	267.2/89.76	2
CD0.5-H240W84	821.8/95.17	95.17	253.5/86.49	268.7/90.27	3
CD0.5-H300W84	821.8/95.17	95.17	253.7/86.49	269.2/90.43	3
CD0.5-H60W112	807.9/93.55	93.55	251.1/83.55	263.1/88.36	2
CD0.5-H120W112	807.9/93.55	93.55	249.9/83.55	262/88	2
CD0.5-H180W112	807.9/93.55	93.55	249.6/83.55	263.8/88.61	2
CD0.5-H240W112	807.9/93.55	93.55	249.5/83.55	264.5/88.85	3
CD0.5-H300W112	807.9/93.55	93.55	249.6/83.55	265.1/89.06	3
CD0.5-H60W140	794/91.94	91.94	247.7/81.62	261.1/87.71	2
CD0.5-H120W140	794/91.94	91.94	246.3/81.62	259.6/87.2	4
CD0.5-H180W140	794/91.94	91.94	245.9/81.62	261.1/87.69	4
CD0.5-H240W140	794/91.94	91.94	245.8/81.62	261.3/87.78	5
CD0.5-H300W140	794/91.94	91.94	245.8/81.62	261.6/87.89	5
CD0.5-H60W280	724.4/83.88	84.87	226.4/83.88	250.4/84.1	5
CD0.5-H120W280	724.4/83.88	84.87	225.2/83.88	247.6/83.19	6
CD0.5-H180W280	724.4/83.88	84.87	224.8/83.88	247.6/83.18	6
CD0.5-H240W280	724.4/83.88	84.87	224.3/83.88	247.7/83.21	6
CD0.5-H300W280	724.4/83.88	84.87	225.2/83.88	247.6/83.19	6
CD1-H60W28	835.9/96.79	96.79	258.1/90.13	266.7/89.6	2
CD1-H120W28	835.9/96.79	96.79	257.8/90.13	267.8/89.94	2
CD1-H180W28	835.9/96.79	96.79	257.7/90.13	270.2/90.77	2
CD1-H240W28	835.9/96.79	96.79	257.8/90.13	272.9/91.69	3
CD1-H300W28	835.9/96.79	96.79	258/90.13	273.1/91.74	3
CD1-H60W56	808.2/93.59	93.59	248.9/81.12	256.6/86.19	2
CD1-H120W56	808.2/93.59	93.59	248.2/81.12	257.5/86.48	2
CD1-H180W56	808.2/93.59	93.59	248/81.12	259.3/87.11	2
CD1-H240W56	808.2/93.59	93.59	248.2/81.12	263.5/88.5	2
CD1-H300W56	808.2/93.59	93.59	248.5/81.12	263.8/88.63	3
CD1-H60W84	780.5/90.38	90.38	240.5/73.76	248.5/83.47	2
CD1-H120W84	780.5/90.38	90.38	239.4/73.76	249.1/83.69	2
CD1-H180W84	780.5/90.38	90.38	239.4/73.76	249.1/83.69	2
CD1-H240W84	780.5/90.38	90.38	239.3/73.76	254.1/85.34	3
CD1-H300W84	780.5/90.38	90.38	235.5/73.76	254.2/85.37	3
CD1-H60W112	752.9/87.18	87.18	233/68.41	241.3/81.06	4
CD1-H120W112	752.9/87.18	87.18	231.5/68.41	241.8/81.22	4
CD1-H180W112	752.9/87.18	87.18	231.2/68.41	243.8/81.89	4
CD1-H240W112	752.9/87.18	87.18	231.2/68.41	245.6/82.49	5
CD1-H300W112	752.9/87.18	87.18	231.4/68.41	245.2/82.37	5
CD1-H60W140	725.2/83.97	83.97	226/65	234.9/78.9	4
CD1-H120W140	725.2/83.97	83.97	224.2/65	235.1/78.96	4
CD1-H180W140	725.2/83.97	83.97	223.7/65	237.1/79.64	4
CD1-H240W140	725.2/83.97	83.97	223.6/65	239.2/80.34	4
CD1-H300W140	725.2/83.97	83.97	223.7/65	237.9/79.91	5
CD1-H60W280	586.8/67.95	69.55	183.9/67.95	196.9/66.14	6
CD1-H120W280	586.8/67.95	69.55	182.5/67.95	194.4/65.32	6
CD1-H180W280	586.8/67.95	69.55	182/67.95	194.8/65.44	6
CD1-H240W280	586.8/67.95	69.55	182.5/67.95	194.9/65.47	6
CD1-H300W280	586.8/67.95	69.55	182.2/67.95	194.9/65.48	6
CD1.5-H60W28	822.3/95.22	95.22	253.1/85.38	260.6/87.55	2
CD1.5-H120W28	822.3/95.22	95.22	252.8/85.38	261.6/87.87	2
CD1.5-H180W28	822.3/95.22	95.22	252.8/85.38	263.6/88.55	2
CD1.5-H240W28	822.3/95.22	95.22	253/85.38	267.4/89.81	2
CD1.5-H300W28	822.3/95.22	95.22	253.2/85.38	268.4/90.14	3
CD1.5-H60W56	781/90.44	90.44	238.9/72.28	244.7/82.19	2
CD1.5-H120W56	781/90.44	90.44	238.2/72.28	246/82.64	2
CD1.5-H180W56	781/90.44	90.44	238.2/72.28	247.5/83.13	2
CD1.5-H240W56	781/90.44	90.44	238.6/72.28	250.5/84.16	2
CD1.5-H300W56	781/90.44	90.44	239.1/72.28	254.1/85.35	3
CD1.5-H60W84	739.7/85.66	85.66	227.1/61.96	231/77.58	2
CD1.5-H120W84	739.7/85.66	85.66	225.1/61.96	232.9/78.22	2
CD1.5-H180W84	739.7/85.66	85.66	224.9/61.96	234.6/78.81	2
CD1.5-H240W84	739.7/85.66	85.66	225.3/61.96	237.5/79.79	2
CD1.5-H300W84	739.7/85.66	85.66	225.9/61.96	239.6/80.48	2
CD1.5-H60W112	698.4/80.88	80.88	215.2/54.79	218.6/73.42	4
CD1.5-H120W112	698.4/80.88	80.88	213.1/54.79	220.6/74.11	4
CD1.5-H180W112	698.4/80.88	80.88	212.9/54.79	222.7/74.81	4
CD1.5-H240W112	698.4/80.88	80.88	213.1/54.79	225.2/75.64	4
CD1.5-H300W112	698.4/80.88	80.88	213.5/54.79	226.1/75.95	2
CD1.5-H60W140	657.2/76.1	76.10	203.9/50.34	206.7/69.44	4
CD1.5-H120W140	657.2/76.1	76.10	200.4/50.34	208.7/70.09	4
CD1.5-H180W140	657.2/76.1	76.10	202.9/50.34	210.5/70.7	4
CD1.5-H240W140	657.2/76.1	76.10	203.3/50.34	212.8/71.47	4
CD1.5-H300W140	657.2/76.1	76.10	203.6/50.34	214.2/71.95	4
CD1.5-H60W280	450.8/52.2	54.05	140/52.2	144.2/48.42	6
CD1.5-H120W280	450.8/52.2	54.05	140.2/52.2	144.5/48.53	6
CD1.5-H180W280	450.8/52.2	54.05	139.9/52.2	144.7/48.59	6
CD1.5-H240W280	450.8/52.2	54.05	141/52.2	144.5/48.55	6
CD1.5-H300W280	450.8/52.2	54.05	139.9/52.2	144.8/48.63	6
CD2-H60W28	808.8/93.66	93.66	248.2/80.77	254.7/85.55	2
CD2-H120W28	808.8/93.66	93.66	247.8/80.77	255.7/85.91	2
CD2-H180W28	808.8/93.66	93.66	247.9/80.77	257.5/86.48	2
CD2-H240W28	808.8/93.66	93.66	248.1/80.77	261.1/87.7	2
CD2-H300W28	808.8/93.66	93.66	248.5/80.77	264/88.68	3
CD2-H60W56	754.1/87.33	87.33	228.6/63.93	232.4/78.06	2
CD2-H120W56	754.1/87.33	87.33	228.6/63.93	233.9/78.55	2
CD2-H180W56	754.1/87.33	87.33	228.6/63.93	234.4/78.75	2
CD2-H240W56	754.1/87.33	87.33	229.5/63.93	236.7/79.5	2
CD2-H300W56	754.1/87.33	87.33	229.1/63.93	241.7/81.18	2
CD2-H60W84	699.4/80.99	80.99	204.9/51.22	212.8/71.48	4
CD2-H120W84	699.4/80.99	80.99	211.9/51.22	215/72.22	2
CD2-H180W84	699.4/80.99	80.99	210.9/51.22	215.9/72.52	2
CD2-H240W84	699.4/80.99	80.99	206.1/51.22	218.5/73.41	2
CD2-H300W84	699.4/80.99	80.99	206.8/51.22	220.3/73.99	5
CD2-H60W112	644.7/74.65	74.65	188/42.83	194.5/65.33	4
CD2-H120W112	644.7/74.65	74.65	193/42.83	196.5/66.02	4
CD2-H180W112	644.7/74.65	74.65	192.7/42.83	198.4/66.64	4
CD2-H240W112	644.7/74.65	74.65	187/42.83	201.5/67.68	4
CD2-H300W112	644.7/74.65	74.65	196.2/42.83	201.8/67.79	4
CD2-H60W140	589.9/68.31	68.31	173.9/37.75	177.5/59.63	4
CD2-H120W140	589.9/68.31	68.31	170.4/37.75	179.6/60.34	4
CD2-H180W140	589.9/68.31	68.31	166.8/37.75	181.4/60.95	4
CD2-H240W140	589.9/68.31	68.31	172.5/37.75	184.4/61.93	4
CD2-H300W140	589.9/68.31	68.31	177.6/37.75	183.7/61.72	5
CD2-H60W280	316.3/36.63	38.37	97.8/36.63	98.3/33.03	6
CD2-H120W280	316.3/36.63	38.37	89.9/36.63	98.3/33.02	6
CD2-H180W280	316.3/36.63	38.37	97.2/36.63	98.3/33.03	6
CD2-H240W280	316.3/36.63	38.37	98.1/36.63	98.3/33.03	6
CD2-H300W280	316.3/36.63	38.37	95.9/36.63	98.3/33.03	6
